# Activatable Nanoparticles: Recent Advances in Redox-Sensitive Magnetic Resonance Contrast Agent Candidates Capable of Detecting Inflammation

**DOI:** 10.3390/ph14010069

**Published:** 2021-01-16

**Authors:** Chukwuazam Nwasike, Erin Purr, Eunsoo Yoo, Jaspreet Singh Nagi, Amber L. Doiron

**Affiliations:** 1 Department of Biomedical Engineering, Binghamton University (SUNY), Binghamton, NY 13902, USA; cnwasik1@binghamton.edu (C.N.); epurr1@binghamton.edu (E.P.); 2 Department of Otolaryngology-Head & Neck Surgery, University of Pennsylvania, Philadelphia, PA 19104, USA; Eunsoo.Yoo@pennmedicine.upenn.edu; 3 Department of Electrical and Biomedical Engineering, University of Vermont, Burlington, VT 05405, USA; jaspreet-singh.nagi@uvm.edu

**Keywords:** MR contrast agents, responsive, relaxation, redox-activatable

## Abstract

The emergence of activatable magnetic resonance (MR) contrast agents has prompted significant interest in the detection of functional markers of diseases, resulting in the creation of a plethora of nanoprobes capable of detecting these biomarkers. These markers are commonly dysregulated in several chronic diseases, specifically select cancers and inflammatory diseases. Recently, the development of redox-sensitive nanoparticle-based contrast agents has gained momentum given advances in medicine linking several inflammatory diseases to redox imbalance. Researchers have pinpointed redox dysregulation as an opportunity to use activatable MR contrast agents to detect and stage several diseases as well as monitor the treatment of inflammatory diseases or conditions. These new classes of agents represent an advancement in the field of MR imaging as they elicit a response to stimuli, creating contrast while providing evidence of biomarker changes and commensurate disease state. Most redox-sensitive nanoparticle-based contrast agents are sensitive to reductive glutathione or oxidative reactive oxygen species. In this review, we will explore recent investigations into redox-activatable, nanoparticle-based MR contrast agent candidates.

## 1. Introduction

Magnetic resonance (MR) imaging is one of the most powerful diagnostic tools available in clinical use [1,2,3,4]. Almost a third of all MR scans are performed using an MR contrast agent, which can be administered orally, intravenously, or through inhalation in order to enhance resulting diagnostic images [2,5,6,7]. These agents can alter the magnetic moment of ^1^H in a local tissue microenvironment to either increase or decrease the MR signal on images, depending on the contrast agent used and the imaging parameters [8,9,10]. Amongst several classes of agents, nanoparticle-based contrast agents have been investigated extensively due to several unique advantages [11]. One of these advantages is the ability of nanoparticles to accumulate in tumor sites via the enhanced permeability and retention (EPR) effect [12,13,14]. Additionally, the large surface area of nanoparticles allows for increased reactivity and provides room for functionalization with specific moieties to improve the targeting of disease sites or introduce other functionalities [11]. Nanoparticle-based contrast agents are generally classified as either T_1_ or T_2_ agents, depending on whether they have a stronger effect on longitudinal (T_1_) or transverse (T_2_) relaxation [15,16,17]. Another class of agents, known as chemical exchange saturation transfer (CEST), is emerging as an MR contrast approach capable of significantly improving diagnostic MR [18,19]. These relatively new CEST-based agents work by exchanging their presaturated exchangeable protons with those of bulk water. The MR contrast is switched “ON” and “OFF” via irradiation with radiofrequency (RF) pulses [11]. 

As MR scans move towards functional detection, researchers have investigated activatable nanoparticle-based MR contrast agents as viable candidates to detect several biological processes. These new types of agents elicit a response to specific stimuli, thereby reporting the presence of phenomena such as an acidic pH, active enzymes, or redox environment [20,21]. Ideally, the contrast agent will go from a non-detectable OFF state to a detectable ON state after interaction with the stimuli of interest, which acts as the activation trigger [5]. The advantage of using an activatable contrast agent is to gain insight on dynamic biological processes and obtain functional diagnostic information in real time to help determine disease risks and severity staging [21,22]. Unlike most contrast agents of the past, activatable nanoparticle-based contrast agents are “not always on” [11]. One of the major issues with those contrast agents is the continuous emission of signal regardless of the proximity and interaction with target tissues and cells. This behavior results in a low target-to-background ratio, making it difficult to detect the target of interest [11]. Activatable nanoparticle-based contrast agents reverse these behaviors by shielding MR contrast until nanoparticles interact with the activation trigger. Specific interactions between activating factors at target sites and nanoparticles result in the activation of MR signal and the maximization of signal at target sites compared to “always on” contrast agents. This process improves the specificity and sensitivity of the imaging nanoprobe.

Most activatable nanoparticle-based contrast agent candidates in the pipeline are often designed to detect biochemical processes in cancers and other inflammatory diseases. These candidates detect changes in the pH of the tumor microenvironment of various cancers, matrix metalloproteinase (MMP) or caspase dysregulation, and redox imbalance. Detecting tissue or cell redox is particularly interesting because it reports changes in chemical metabolic processes as well as changes characteristic of chronic inflammatory diseases. Under healthy circumstances, intracellular and extracellular redox environments are tightly regulated in tissues [23]. The dysregulation of antioxidants such as glutathione results in changes in redox state which are usually associated with physiological and pathophysiological disorders. Studies have pinpointed altered redox as a culprit in the increased production of reactive oxygen species (ROS) [24,25,26]. ROS play an important role in cellular proliferation, differentiation, apoptosis, and senescence. However, excessive ROS production has been linked to tissue injury, endothelial dysfunction, and poor inflammatory response [27,28,29]. Researchers have capitalized on the role of redox in inflammatory diseases by creating several contrast agents that interact with biothiols and free radicals to activate or release encapsulated contrast agents in vitro and in vivo [30]. These contrast agents report on the abnormal production and expression of redox molecules. In this review, we explore the most recent advances in redox-sensitive contrast agent candidates capable of detecting inflammation. Each agent is classified according to the activating factor, while the mechanism and strategies underlying their activation will be discussed in detail within each section.

## 2. Glutathione Activatable Nanoparticle-Based Contrast Agents

Glutathione (GSH) is the most abundant low-molecular-weight thiol and has been investigated extensively as an antioxidant [31]. Glutathione disulfide (GSSG) is produced during thiol oxidation and promotes the formation of disulfide bonds, which are critical to the formation of proteins’ secondary, tertiary, and quaternary structures. Disulfide bonds are considered great linkers because they are unstable and easily broken down by GSH [31,32]. The interactions between GSH and disulfide bonds make GSH a great target for redox-based nanoparticles. Some of these particles are turned OFF with surface coatings linked by disulfide bonds. In the presence of GSH, the bond will be cleaved, allowing or restricting access to water resulting in contrast. The presence of glutathione is key to biological functions as it aids detoxification and protects against oxidative damage.

### 2.1. MR Specific Glutathione Responsive Contrast Agents

Reductive environments are very common in inflammatory diseases often diagnosed with MR scans due to changes in the level of glutathione produced at the disease site. Some of these contrast agent candidates are specific to MR scans and not investigated for multimodal imaging. One of these agents was created by Ye and coworkers [30]. The group created a contrast agent that consists of gadolinium (Gd (III); Gd^3+^) chelates conjugated to an acyclic precursor that self-assembles when exposed to a thiol reducing environment [30]. The Gd (III) chelates undergo intramolecular macrocyclization to form hydrophobic macrocycles that then self-assemble into Gd (III) nanoparticles (GdNPs). The synthesis of the probe led to the development of two isomers, 1-I and 1-II, which were confirmed as isomers when the chiral center was removed via oxidation to form probe 1-O. Dynamic light scattering (DLS) confirmed both isomers, and the oxidation compounds were able to successfully self-assemble into GdNPs when exposed to a reducing environment both in buffer and human breast cancer cells MDA-468. Furthermore, 1-I, 1-II, and 1-O all had r_1_ relaxivity (r_1_) similar to those of regular Gd (III) chelates. After disulfide reduction, all probes showed an increase in r_1_ at a field strength of 0.5 T, specifically 1.5-fold (1-I), 1.6-fold (1-II), and 1.4-fold (I-O) increases. Similar r_1_ increases were also observed at field strengths of 1.5 T and 3.0 T. Overall, 1-O showed the highest r_1_ relaxivity across all field strengths: 34.2 mM^−1^s^−1^ at 0.5 T, 31.8 mM^−1^s^−1^ at 1.5 T, and 21.0 mM^−1^s^−1^ at 3 T. Interestingly, the r_1_ relaxitivity of 1-O was eight times greater at 3T than the clinically used Gd^3+^ contrast agent Dotarem. Incubation with the biothiol glutathione showed T_1_ values of 17 ± 4 ms in 1-I and 74 ± 5 ms in 1-II. Similar levels of T_1_ values were also achieved when the GdNPs were incubated with HeLa cells, showing that the nanoparticle can activate at biologically relevant levels. This study presents a straightforward method to functionalize GdNP to detect biological redox, specifically GSH. The macrocyclization chemistry-mediated self-assembly concept should be investigated further to understand the potential benefits to redox activatable nanoparticle-based contrast agents as well as the potential roles of disulfide reduction in the enhancement of relaxivity.

**Figure 1:**
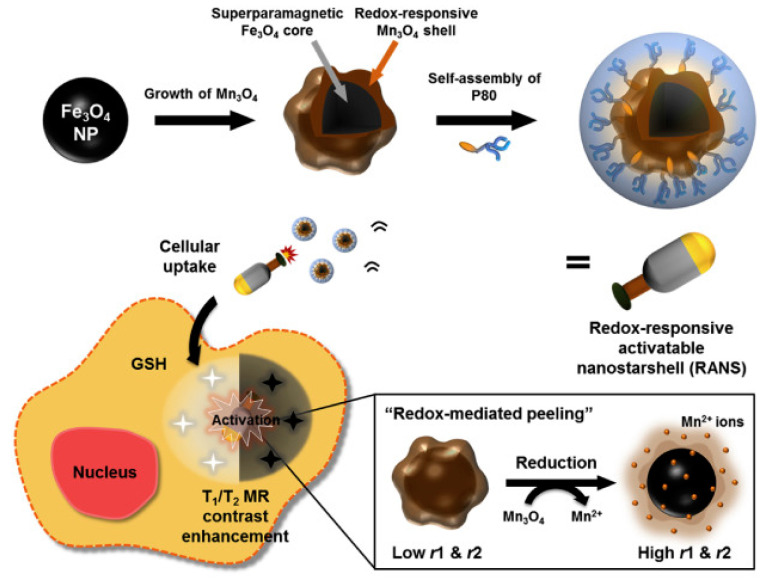
Redox-responsive activatable nanostarshell (RANS). GSH: Glutathione. Reprinted (adapted) with permission from [34]—Published by Elsevier.

Given the promising results obtained from redox functionalized Gd (III) chelate particles to detect T_1_ signals, Li et al. fabricated glutathione-activated MR nanoprobes that detect cancerous cells utilizing the distance change between T_1_ and T_2_ MR contrast agents [33]. The nanoparticle was comprised of superparamagnetic iron oxide (SPIOs; Fe_3_O_4_), gadolinium, polyethylene glycol (PEG) and cystamine (SS) (Fe_3_O_4_-SS-PEG Gd_2_O_3_). Cystamine was used to link the gadolinium and iron oxide nanoparticles because of its disulfide linkage capability. Iron oxide interfered with the T_1_ effects of gadolinium when in close proximity in the nanoprobe, thereby turning off the T_1_ contrasting ability. In the presence of GSH, cystamine was cleaved, allowing T_1_ enhancement by gadolinium. To investigate cystamine cleavage and the enhancement of T_1_ signals, Fe_3_O_4_-SS-PEG Gd_2_O_3_ was exposed to different molecules such as glucose, fructose, glutathione, alanine, and histidine. MR scans showed significant T_1_ signal enhancement (4×) when particles were exposed to glutathione compared to other biomolecules. Having confirmed the glutathione-mediated activation of T_1_ signals, the concentration-dependent effects of glutathione on Fe_3_O_4_-SS-PEG Gd_2_O_3_ were further investigated. The glutathione activation of nanoparticles appeared to plateau at 3 mM concentration and did not improve signal at higher concentrations. Fe_3_O_4_-SS-PEG Gd_2_O_3_ was functionalized with nucleolin-targeted DNA aptamer (AS1411- Fe_3_O_4_-SS-PEG Gd_2_O_3_) and exposed to human kidney cancer cells (786-0 cells) for further studies into the in vitro activation of T_1_ signals. The results showed that AS1411-Fe_3_O_4_-SS-PEG Gd_2_O_3_ specifically bound to nucleolin, which is overexpressed in cancer cells, facilitating uptake of the nanoprobe and the subsequent cellular activation of T_1_ signals. Strong T_1_ signals were observed 1 h post nanoprobe treatment. Slight toxicity to 786-0 cells was also detected for higher concentrations, but this was not statistically significant. After Li et al.’s success with dual T_1_ and T_2_ agents, Kim and colleagues investigated redox induced dual simultaneous T_1_/T_2_ MR contrast agent activation [34]. Unlike Li and Co., this research group performed a more in-depth analysis of an activatable T_1_/T_2_ dual-mode MR probe consisting of an Fe_3_O_4_ hybrid nanocrystal core coated with trimanganese tetraoxide (Mn_3_O_4_; manganese—Mn) (Figure 1). The nanocrystals were synthesized via a seed-mediated growth method and encapsulated within amphiphilic PEG derivatives. These redox-responsive activatable nanostarsells (RANS) were activated by glutathione in an intracellular reducing environment. The GSH activation of RANS resulted in an r_1_ relaxivity of 16.1 mM^−1^s^−1^ and an r_2_ relaxivity (r_2_) of 258.6 mM^−1^s^−1^, both significantly higher than in the ‘OFF” state of 2.4 mM^−1^s^−1^ and 92.2 mM^−1^s^−1^, respectively. The T_1_/T_2_ MR contrasts of the RANS were investigated in vitro using human gastric cancer cells (MKN-45). Cells treated with RANS showed a 187.9% contrast increase in the T_1_-weighted image and a 338.5% contrast increase in the T_2_-weighted image when compared to non-treated cells. RANS were tested in vivo in MKN-45 tumor-bearing mice. The results showed that nanoparticles that accumulated in tumors had a 49.8% increase in T_1_ signal intensity and a 63.7% decrease in T_2_ signal intensity 2 h after injection. With these results, the potential of the T_1_/T_2_ dual-mode MR probe was established. 

**Figure 2:**
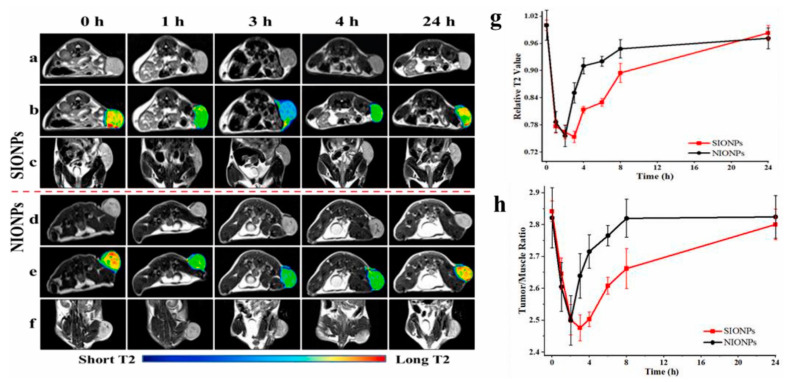
In vivo T_2_-weighted imaging (**a**,**d**), pseudo-color images (**b**,**e**) and coronal magnetic resonance (MR) images (**c**,**f**) of C6 tumor-bearing mice. (**g**,**h**) Quantification of signals. NIONPs: Non-sensitive superparamagnetic Fe_3_O_4_ nanoparticles; SIONPs: Sensitive superparamagnetic Fe_3_O_4_ nanoparticles. Reprinted (adapted) with permission from [35]—Published by Elsevier.

Recent investigation into GSH activatable contrast agents aimed to develop a novel contrast agent based on sensitive superparamagnetic Fe_3_O_4_ nanoparticles (SIONPs) with enhanced MR signal for the specific diagnosis of glioma [35]. Currently, the only available method to diagnose glioma is biopsy, which is often invasive and risky. The contrast agent core SIONPs were encapsulated with methoxy-poly-(ethylene glycol)-S-S-hexadecyl (mPEG-S-S-C16), which acted as a firm coating over the nanoparticles to ensure efficient delivery of the contrast agent to the target tumor site. The interaction of the contrast agent with the GSH at the tumor site resulted in the enhancement of the MR signal and helped in the specific diagnosis of glioma. The T_2_-weighted MR images taken at 3T indicated that the r_2_ relaxivity of SIONPs and non-sensitive superparamagnetic Fe_3_O_4_ nanoparticles (NIONPs) was similar in the absence of GSH. However, the introduction of GSH resulted in a 1.83-fold increase in r_2_ for SIONPs compared to no significant change detected in NIONPs. These changes in r_2_ values demonstrated the GSH-dependent enhancement of the MR signals using SIONPs. The GSH-dependent behavior of the SIONPs was tested in vivo using a subcutaneous tumor model. T_2_-weighted enhancement was detected in models treated with SIONPs and NIONPs. In vivo activation of the T_2_ signal was highest in tumor regions treated with SIONPs, with a significant loss in signal intensity compared to NIONPs (Figure 2). The results indicate that GSH-dependent SIONP-based MR contrast agents can be used in the formation of a non-invasive differential diagnosis method for glioma. Despite promising results, the primary concern with these contrast agent candidates releasing metals such as manganese remains biocompatibility, which might raise toxicity issues. Further studies into biocompatibility are required to better understand the clinical potential of these contrast agent candidates.

### 2.2. Multimodal Glutathione Responsive Contrast Agents

Several researchers have advanced glutathione-mediated redox responsive nanoparticle-based contrast agents by formulating multimodal agents. These agents can provide functional diagnostic information from multiple imaging modalities at the same time. One such agent was formulated by Zheng et al. in 2016. This research group investigated trimodal efficacy by fabricating a redox-sensitive fluorescence/^19^F-MRS/^1^H-MR triple-functional nanoprobe [36]. The nanoprobe was comprised of gadolinium chelate linked with a quenched amino oxyluciferin fluorophore and a ^19^F-bearing moiety. Amino oxyluciferin was attached to the ^19^F-bearing moiety through a disulfide linker that can be cleaved by biothiols to activate the particle. In the presence of biothiols such as GSH, the nanoprobe enhanced T_1_ signals significantly. The relaxivity for activated nanoprobes was 23.6 ± 0.2 mM^−1^s^−1^ compared to inactivated nanoprobes, which had r_1_ values of 5.4 ± 0.3 mM^−1^s^−1^. Having established a nanoprobe sensitive to GSH, HeLa cells were used to investigate the in vitro activation of the nanoprobes. T_1_ values of the activated nanoprobes were much higher (at least 175 ms) than the control (about 120 ms), which lacked the disulfide linker. This study provided early stage evidence that the redox-sensitive fluorescence/^19^F-MRS/^1^H-MR triple-functional nanoprobe is capable of inducing MR contrast via disulfide bond cleavage despite roles in other image modalities.

Around the same time, Gallo and coworkers created manganese dioxide (MnO_2_) nanosheets that could be activated as T_1_ contrast agents and deliver carbon quantum dots (CQDs) under certain physiological conditions to act as targeted, multimodal MR/optical probes [37]. In the MnO_2_ nanosheets, Mn^4+^ showed weak paramagnetic properties, and the fluorescence of the CQDs was quenched due to their close proximity to the sheets. When exposed to GSH or H_2_O_2_, MnO_2_ was reduced to Mn^2+^, which is a highly paramagnetic T_1_ contrast agent. The nanosheet dissolved and released the CQDs, allowing an optical signal to be generated. The nanosheets were created entirely in water using glucose as a mild reducing agent and ligand, removing the need for harsh chemicals and organic solvents used to create other contrast agents. When introduced into A549 human epithelial cells, the MnO_2_-CQDs showed no cytotoxicity. A 26.03% increase in signal intensity in T_1_—weighted contrast was seen 12 min after the addition of the nanosheets. Gallo and coworkers believe that this will create an effective method to improve the specificity of MR and that the simple, eco-friendly preparation method can be scaled up to industrially relevant quantities.

The most recent advancement of GSH-mediated trimodal imaging investigated the use of a GSH sensitive, liver-targeted probe for the real-time visualization of hepatic GSH levels for the diagnosis and prognosis of acute hepatitis. Hu and coworkers aim to create a better method for measuring hepatic GSH levels than existing GSH-responsive fluorescent imaging probes, which are limited by the low penetration depth of light in the liver [38]. GdNPs-Gal were formed via the molecular coassembly of a GSH-responsive Gd(III) MR probe and a liver targeting probe (1-Gal). The GdNPs-Gal showed a high r_1_ relaxivity with low fluorescence and ^19^F-MRS signal. Once exposed to GSH, disulfide bonds were reduced and the NPs disassembled, reducing r_1_ relaxivity and ^1^H-MR contrast but increasing fluorescence and ^19^F-MRS signal. In vitro, the initial r_1_ relaxivity of GdNPs-Gal was 15.6 ± 0.3 mM^−1^s^−1^ at 0.5 T, which was about three times higher than Dotarem. After the introduction of GSH, the r_1_ relaxivity declined to 5.3 ± 0.1 mM^−1^s^−1^. The T_1_ relaxation time increased from 382 ms to 838 ms after incubation with GSH for 1 h, in a concentration dependent manner. GdNPs-Gal were injected in vivo into lipopolysaccharide-induced inflammatory mice. A bright T_1_—weighted MR contrast was observed in the liver 1 h after injection, showing the nanoparticles accumulated in the liver. After 1 h, a rapid decline in MR contrast was detected because of reduced r_1_ relaxivity triggered by cleavage and the activation of nanoparticles. In contrast, GSH-inert GdNPs-Gal-control showed an increase in T_1_ weighted MR contrast that remained high for more than 8 hrs. With these results obtained, Hu et al. showed that their GdNPs-Gal could effectively target the liver and be activated upon reduction by elevated levels of GSH. Although GSH has shown promising results both in vitro and in vivo, none of these nanoparticles are approved by the food and drug administration (FDA), which highlights the challenges of translating nanoparticle-based contrast agents from bench to bedside (Table 1).

## 3. ROS Activatable Nanoparticle-Based Contrast Agents

Reactive oxygen species are chemical species produced as byproducts of normal cellular metabolism [23]. Even though they are highly unstable, very reactive and short lived, they are capable of independent existence. At low to moderate levels, they play important roles in various physiological conditions such as redox regulation, immune response, mitogenic response, and cellular signaling pathways [23]. However, high levels of ROS result in oxidative stress, which can damage cells, tissues, and biomolecules due to the poor regulation of enzymatic and non-enzymatic antioxidants [28,29]. Several inflammatory diseases such as atherosclerosis, rheumatoid arthritis, cancers, diabetes mellitus, and neurodegenerative diseases are characterized by ROS dysfunction [39,40]. Increased levels of ROS are associated with the pathogenesis of many diseases, and a non-toxic agent activated by ROS could improve not only disease diagnosis, but also treatment monitoring of disease over time and therefore patient outcomes. Therefore, researchers have actively explored the formulation of nanoparticle-based contrast agents sensitive to ROS to detect inflammatory diseases. These nanoparticles can be oxidized by different species of ROS, thereby activating the contrast agent’s properties. 

### 3.1. Peroxide Sensitive Agents

Peroxides are perhaps the most targeted species of ROS by nanomaterial scientists. Amongst peroxides, hydrogen peroxide (H_2_O_2_) has been targeted and investigated extensively, partly because it is the second most abundant species of ROS in the body [41]. It is also actively involved in the synthesis and generation of other species of ROS [42,43]. Given the importance of peroxides, researchers have developed contrast agents capable of detecting peroxide. One of these investigations was conducted by Fu and coworkers, who created a novel ^19^F polymeric imaging agent that can be activated in the presence of ROS [44]. The nanoparticles consisted of thioether- and fluorine-containing methacrylate monomers synthesized via atom transfer radical polymerization. The polymers self-assembled into nanoparticles with a strong hydrophobic core in an aqueous solution. In the core, the movement of the fluorinated segments was limited, shortening the T_2_ relaxation times and reducing the ^19^F NMR signal. In the presence of ROS, the hydrophobic thioether groups were oxidized and the nanoparticles disaggregated. Once disassembled, the T_2_ relaxation times of ^19^F were lengthened and the NMR signal increased. In addition, the researchers also made the nanoparticles sensitive to pH, increasing the change in intensity when the agents were exposed to ROS in a mildly acidic environment. A polymer with 4.7 weight percent (wt%) fluorine was treated with H_2_O_2_ and after 20 h of oxidation, the ^19^F T_2_ relaxation time was lengthened to 34 ms, causing a brighter image. Before oxidation, no ^19^FMR signal was observed. The ROS/pH dual responsive ^19^F MR agent showed increased signal when exposed to either low pH or H_2_O_2_, and an even greater increase in signal when exposed to both. However, at lower levels of ROS, they observed a slower oxidation rate and the conversion of thioether groups was limited to 23%. Moving forward, the research group plan to increase the sensitivity of the polymers to ROS before starting in vivo experiments.

A peroxide-sensitive, nanoparticle-based contrast agent was also created by Rodríguez and colleagues. Their research group developed a peroxidase-sensitive T_1_ MR contrast agent comprised of a macrocyclic Gd-DOTAGA core with 5-hydroxytryptamide (5-HT) moiety [45]. The contrast agent was designed to overcome current limitations associated with nanoparticle-based contrast agents such as poor solubility in aqueous media and poor release of Gd (III) in vivo. Inflammation in the lungs proceeds when leukocytes (neutrophils, eosinophils, and alveolar macrophages) are activated, and they release oxidative enzymes such as myeloperoxidase (MPO) and eosinophil peroxidase (EOP) that can produce ROS. In the presence of H_2_O_2_, the oxidases can oxidize the Gd^3+^ complexes bearing 5-HT or related moieties to form oligomers or adducts with plasma and matrix proteins. Thus, the r_1_ relaxivity of Gd^3+^ is increased due to the increased rotational correlation time and retention by its increased size. In horseradish peroxidase/hydrogen peroxide conditions, Gd-5-HT-DOTAGA showed 1.7-fold increased r_1_ in 20 min; however, Gd-T-DOTAGA showed no change. Gd-5-HT-DOTAGA also showed 40% increased r_1_ when it was incubated with 0.3 U MPO in lung tissue homogenate, while there was no change with Gd-T-DOTAGA in the same condition. For in vivo T_1_-weighted imaging, bleomycin was injected into the lungs of C57BL/6 mice via intratracheal instillation, resulting in a strong inflammatory response causing fibrosis. Gd-5-HT-DOTAGA triggered strong T_1_ MR signal in the lungs of bleomycin-injured mice but not in uninjured mice. With the observed results, Gd-5-HT-DOTAGA, with improved solubility and stability, provided the potential to assess specific detection of lung inflammation by MR.

**Figure 3:**
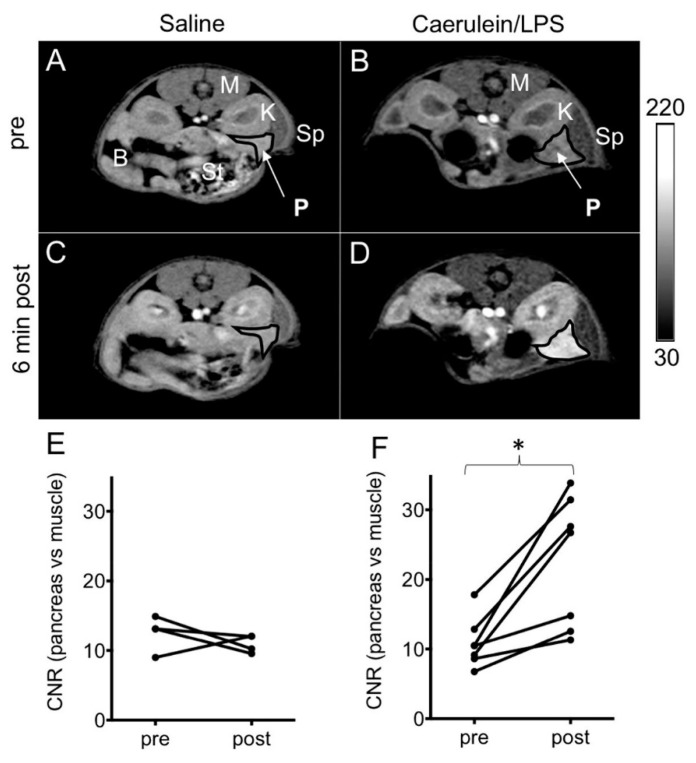
*(***A**–**D**) T_1_-weighted images of saline and caerulein/LPS-treated mice scanned pre- and 6 min post injection of 0.2 mmol/kg Fe^2+^-PyC3A. (**E**,**F**) Contrast to noise ratio (CNR) in pancreas vs. muscle before and 6 min after contrast injection; * *p* < 0.0001. Reprinted (adapted) with permission from [46]—Published by American Chemical Society (ACS).

Further, Wang and colleagues expanded their knowledge of peroxide-sensitive contrast agents by introducing a redox-active iron complex, Fe-PyC3A, that could switch between Fe^3+^-PyC3A and Fe^2+^-PyC3A through oxidation by hydrogen peroxide [46]. The researchers hypothesized that the relaxivity difference between Fe^2+^ and Fe^3+^ oxidation states is large enough to be detectable in vivo with a standard dose of the Fe^2+^ complex. The r_1_ of Fe^3+^-PyC3A was 10-, 13.3-, and 14.5-fold higher than Fe^2+^-PyC3A in pH 7.4 Tris buffer at 1.4, 4.7, and 11.7 T, respectively. To test the capability of Fe-PyC3A to detect ROS in a murine model, the research group developed a mild edematous pancreatic inflammation and acute inflammation model with caerulein (50 µg/kg in saline) and lipopolysaccharide (10 mg/kg in saline) that can stimulate neutrophil activation and ROS production via intraperitoneal injection. After the injection of Fe^2+^-PyC3A, selective significant signal enhancement was detected from the inflamed pancreas, indicating that the Fe^2+^ complex was rapidly converted to the high relaxivity Fe^3+^ oxidation state in the presence of ROS (Figure 3). Together with other studies reviewed, the versatility of peroxide-activatable nanoparticle-based contrast agents shows great promise in the detection of inflammation. Investigation should be advanced to complex biological systems to better understand efficacy and functionalities.

### 3.2. Nanoparticles Sensitive to Other Species of ROS

**Figure 4:**
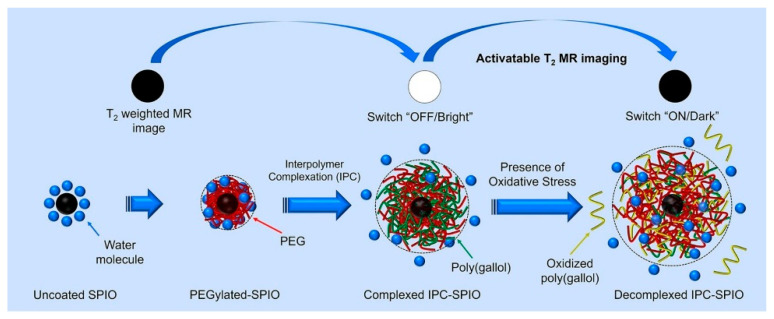
Illustration of activatable interpolymer complex-superparamagnetic iron oxide nanoparticles. SPIO: Superparamagnetic iron oxide nanoparticles; IPC-SPIO: Interpolymer complex-superparamagnetic iron oxide nanoparticles. Reprinted (adapted) with permission from [47]—Published by Elsevier.

In a deviation from more conventional GSH and peroxide-activated contrast agents, our lab’s work by Yoo et al. advanced redox responsive particles by creating activatable interpolymer complex-superparamagnetic iron oxide nanoparticles (IPC-SPIOs) that are sensitive to superoxide [47]. In that preliminary study, IPC-SPIOs were comprised of a superparamagnetic iron oxide core coated with PEG and complexed with poly(gallol) (Figure 4). Complexation, which is the process of hydrogen bonding poly(gallol) to PEG, creates an interpolymer complex that pushes water away from the core, but is sensitive to oxidation. The exposure of IPC-SPIOs to exogenous sources of ROS resulted in ROS scavenging, IPC-SPIO decomplexation, and the activation of T_2_ MR signals as water hydrated the core SPIO. MR scans of ROS-treated complexed IPC-SPIOs (0.25 mg/mL) showed complexed and decomplexed r_2_ values of 7 ± 2 mM^−1^·s^−1^ and 70 ± 10 mM^−1^·s^−1^, respectively. Yoo et al. showed, for the first-time, a T_2_ contrast agent candidate complexed with poly(gallol) activated by oxidation.

With early stage evidence established, Nwasike et al. investigated biologically relevant ROS-mediated IPC-SPIOs activation by macrophages and endothelial cells [48]. Using the 2′,7′-dichlorofluorescin diacetate (DCFDA) ROS detection assay, IPC-SPIOs scavenged over 70% of available ROS in macrophages and human umbilical vein endothelial cells (HUVEC) in vitro with no toxicity. The activation of T_2_ MR signal was observed in macrophages treated with IPC-SPIOs. The T_2_ values of macrophages treated with IPC-SPIOs ranged from 174 ms to 149 ms pre- to post-cellular ROS exposure, indicating about 15% T_2_ signal activation after 6 h. MR contrast detected in macrophages treated with IPC-SPIOs was also 80% higher compared to the controls. Follow-up work with in-depth analysis and functionalization of IPC-SPIOs with a targeting biomolecule to improve nanoparticle uptake to further improve activatable MR contrast is currently underway. 

Other research groups, such as Li et al. have also contributed to the advancement of nanoparticles sensitive to species of ROS not previously explored. In their work, Li and coworkers developed a fluorinated bihydrazide conjugated to gadolinium-tetraazacyclododecanetetraacetic acid (Gd-DOTA) complex to detect hypochlorous acid (HClO) using ^19^F NMR/MR [49]. The ^19^F NMR/MR agent was comprised of Gd-DOTA complex linked with 3,5-bis(trifluoromethyl)benzoic acid via a bihydrazide linker. In the presence of HClO, the bihydrazide linker was selectively oxidized and broken. As a result, the recovery of the T_1_ and T_2_ of the fluorine atoms was significantly increased due to the decreased paramagnetic relaxation enhancement (PRE) effect of Gd-DOTA, responsive to HClO. In the real-time detection of HClO in biological systems, the nanoparticle demonstrated the ability to activate under HClO and image hepatocellular carcinoma human hepatocarcinoma SMMC-7721 cells in nude mice. SMMC-7721 cells were treated with HClO, excess H_2_O_2_, and HClO plus taurine, which is a HClO scavenger. A strong ^19^F NMR signal was observed only from the cells exposed to HClO. In addition, a quantitative analysis indicated the high selectivity to HClO based on the relatively greater signal-to-noise ratio of the HClO exposed group compared to the other groups. In vivo imaging of HClO with nude mice showed that strong ^19^F NMR signal was only detected around the area treated with HClO, indicating outstanding specificity. This investigation provides an alternative to more conventional ROS targets. The ^19^F NMR/MR probe (Gd-DOTA-BTFPH) small molecular conjugate could be used as a potential imaging probe for the selective detection and real-time imaging of hypochlorous acid in organisms.

In a recent communication, Zhou et al. proposed an activatable MR contrast agent capable of detecting the efficacy of radiotherapy in cancer patients [50]. The contrast agent was built as a nanovesicle structure comprised of a triblock poly(ethylene glycol)–poly(propylene sulfide)–poly(ethylene glycol) (PEG–PPS–PEG) amphiphilic copolymer with iron oxide nanoparticles (IO NPs) in the membrane and gadolinium (IO-Gd NV) species on the surface. Inflammation triggers the activatable properties of the nanoparticle as interaction with factors such as ROS results in the self-assembly and disassembly of the IO-Gd NVs. The self-assembly and disassembly process leads to T_1_ OFF-ON effects. To confirm IO-Gd NV ability for stratification, in vivo studies using Balb/c mouse 4T1 tumor models were conducted to investigate the response to radiotherapy. T_1_ mapping results showed different levels of T_1_ signal enhancement in mice treated with radiotherapy only (323 ± 37 ms), radiotherapy and aLy6G (neutrophil depleting antibody; T_1_ = 273 ± 33 ms), radiotherapy and G-CSF (granulocyte colony-stimulating factor; T_1_ = 1372 ± 221 ms), and radiotherapy plus DPI (diphenyleneiodonium, a NADPH oxidase inhibitor; T_1_ = 364 ± 121 ms). The different T_1_ values detected pointed to the different responses to treatment and the effectiveness of radiotherapy in different conditions. This study could be very useful in clinical settings as understanding tumor response to treatment would help quicken decisions and strategies for treatment, thereby saving time and improving patient outcomes (Table 2). 

## 4. Emerging Redox Environment Responsive Contrast Agents

Redox-responsive agents based on unique bioprocesses and materials have been investigated by researchers to advance the field from a different perspective. Several years ago, Dunbar and coworkers explored the use of copper as a redox-sensitive MR contrast agent [51]. Even though the relaxivity of copper is lower than that of the available agents, its ability to switch between copper (I) and copper (II) makes it a potential novel activatable contrast agent. The copper(II) macrocycles tested were ([Cu(II)S_en_N_pr_]^2+^, [Cu(II)S_pr_N_en_]^2+^, [Cu(II)S_pr_N_pr_]^2+^) and [Cu(II)S_pr_N_en_(X)_2_]^2+^, where X was replaced by different chemical chains. These copper(II) macrocycles were compared with gadopentetic acid (Gd(DTPA)) and Cu(BF_4_)_2_. The r_1_ relaxivity of ([Cu(II)S_en_N_pr_]^2+^, [Cu(II)S_pr_N_en_]^2+^, [Cu(II)S_pr_N_pr_]^2+^), and [Cu(II)S_pr_N_en_(–(CH_2_)_2_SPh )_2_]^2+^ were 0.27 ± 0.03 mM^−1^·s^−1^, 0.27 ± 0.01 mM^−1^·s^−1^, 0.26 ± 0.03 mM^−1^·s^−1^, and 0.12 ± 0.02 mM^−1^·s^−1^, respectively, whereas the r_1_ relaxivity of the reference compounds Gd(DTPA) and Cu(BF_4_)_2_ were 4.73 mM^−1^·s^−1^ and 0.67 mM^−1^·s^−1^, respectively. The redox cycling results indicated that [Cu(II)S_pr_N_en_(–(CH_2_)_2_SPh )_2_]^2+^ macrocycle was the only one that was reversible and could switch to copper(I) due to an increase in sulfur density. In addition, it was also discovered that the highest re-oxidation efficiency was approximately 85%. The researchers concluded that copper needs to be combined with a compound with higher relaxivity for better results, but their current findings show the potential of copper as a redox-sensitive MR contrast agent. For now, the primary concern with copper would be biocompatibility, which might raise toxicity issues. It might be challenging to balance toxicity while delivering a sufficient amount of copper for MR contrast. Understanding biocompatibility might be key to unlocking this contrast agent candidate. 

Gale and coworkers followed up by investigating the use of a novel Mn-based MR contrast agent that could isomerize between Mn^3+^ and Mn^2+^ using a Janus ligand attached to HBED (N,N′-Bis(2-hydroxybenzyl)ethylenediamine-N,N′-diacetic acid), and BPED (N,N′-bis(2-pyridylmethyl)ethylenediamine-N,N′-diacetate(2-)) known as JED ligand [52]. The function of a Janus ligand is to promote interchange between Mn^3+^ and Mn^2+^ states. Unlike other ligand systems, JED could support redox states by presenting a HBED-type and BPED-type donor set to Mn^3+^ and Mn^2+^, respectively, on oxidation or reduction. The r_1_ values of Mn^3+^- and Mn^2+^-JED in water and plasma indicated that Mn^2+^ had much higher relaxation values than Mn^3+^. For Mn^2+^, the r_1_ values were 3.3 ± 0.006 mM^−1^·s^−1^, 4.3 ± 0.32 mM^−1^·s^−1^, and 2.5 ± 0.008 mM^−1^·s^−1^, whereas Mn^3+^ had r_1_ values of 0.5 ± 0.001 mM^−1^·s^−1^, 0.9 ± 0.002 mM^−1^·s^−1^, and 0.5 ± 0.001 mM^−1^·s^−1^ at 1.4 T, 4.7 T, and 11.7 T, respectively. These results were supported by comparing the T_1_-weighted MR images of Mn-JED and a Gd^3+^-based agent, which indicated that the Mn^2+^-JED solution was much brighter than the Mn^3+^ solution and the difference between the Gd-based agent images was not significant. The r_1_ change at 1.4 T for Mn-JED and Gd-based agents was 380% and 15%, respectively, which doubled when measured in plasma. These current findings indicate that the Janus ligand can provide a new, promising model for developing MR contrast agents. 

## 5. Redox Activatable Combined Diagnostic and Therapeutic Agents

Medical imaging is primarily used to screen, diagnose, and stage disease in patients as well as monitor treatment and investigate relapse in patients. Recently, therapeutics have been gradually incorporating imaging, which is facilitating a new age of image-guided therapy. This new method of therapy is very attractive because it allows for more accurate therapeutic planning and observed therapeutic delivery and efficacy. With the advent of activatable imaging technologies, several drugs have been incorporated into activatable contrast agents to accomplish image-guided therapy. These types of therapies rely on the activation of the contrast agent upon interaction with an activation trigger, which will release drugs as well as MR contrast agents simultaneously. The key advantage of this type of treatment is that it permits functional imaging as treatment is ongoing in a minimally invasive process. 

**Figure 5:**
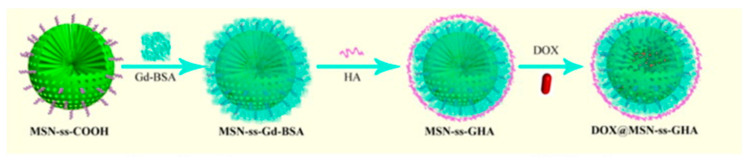
Theranostic redox-responsive mesoporous silica nanoparticles for dual drug delivery and MR imaging. Reprinted (adapted) with permission from [53]—Published by American Chemical Society (ACS).

Several years ago, Chen et al. reported a new multifunctional drug delivery system based on mesoporous silica nanoparticles (MSN) capped by gadolinium-based bovine serum albumin complex (BSA-Gd) and hyaluronic acid (HA) via a reductive-cleavable disulfide bond to release therapeutics and enhance T_1_ MR signal intensity (Figure 5) [53]. The thiol-functionalized MSN (MSN-SH) nanoparticle was synthesized via a one-pot reaction. MSN-ss-COOH was obtained from MSN-SH with 2,2-dithiodipyridine and 3-mercaptopropionic acid treatments via disulfide bonds, which could be broken by a relatively high level of GSH in tumor cells. Subsequently, BSA-Gd complex was conjugated on the surface of MSN-ss-COOH via a carbodiimide-catalyzed amidation reaction, denoted as MSN-ss-Gd-BSA. Finally, MSN-ss-GHA, HA-coated MSN-ss-Gd-BSA nanoparticles, were prepared via amide formation. Anticancer drug doxorubicin (DOX) was also incorporated into the nanocomplex, resulting in DOX@MSN-ss-GHA. In the presence of 10 mM GSH (tumor microenvironment) at pH 5.0, the disulfide bond in MSN-ss-GHA was broken to release the DOX (up to 67.12% with a faster rate due to the faster diffusion rate of DOX at pH 5.0 than pH 7.4). Confocal laser scanning microscopy (CLSM) images showed that MSN-ssGHA has enhanced cell uptake efficacy by 4T1 cells (murine breast cancer cell line) compared to the control MSN-ss-COOH. This is because of the specific binding efficacy of the HA component with the overexpressed CD44 receptor. MSN-ss-GHA enhanced T_1_ signals with an r_1_ value of 17.38 mM^−1^s^−1^ compared to commercial Gd-DTPA contrast agents (4.39 mM^−1^s^−1^). Overall, the great efficacy shown by MSN-ss-GHA nanoparticles makes it a promising theranostic candidate for cancer detection and therapy.

Subsequently, Zhang and coworkers explored a multimodal nanoparticle-based contrast agent candidate in combination with cancer therapy. The researchers investigated the use of a nanoagent sensitive to redox features of tumors for the precise treatment of cancer using the combined effects of chemotherapy and photothermal therapy (PTT), compatible with MR imaging, fluorescence imaging, and photothermal imaging [54]. The development of such a multi-functional theranostic nanoagent was important for the site-specific and efficient treatment of hypoxic solid tumors. Silicon dioxide (SiO_2_) coated with a gold (Au) shell and a MnO_2_ sheet with doxorubicin hydrochloride as the photothermal nanomaterial and chemotherapeutic carrier, respectively, completed the aptamer (Apt) modified nanoagent (SiO_2_@Au@MnO_2_–DOX/Apt). On interaction with tumors, the nanoagent released Mn^2+^ and DOX simultaneously, leading to an increase in the efficiency of chemotherapy. The presence of Mn^2+^, the decomposition of MnO_2_, and the absorption of near infrared (NIR) light were all monitored with MR, fluorescence imaging, and photothermal imaging, respectively. The redox activated drug release was assessed in vitro using MR and fluorescence imaging. The results indicated that the SiO_2_@Au@MnO_2_–DOX/Apt nanoagent remained stable for over 72 h, and only 10% of the drug leaked from the nanoagent. Further, the nanoagent depicted a 43% sharp increase at 5 h and a 90% sharp increase at 10 h when 1 mM and 10 mM glutathione were added. The MR results indicated that the nanoagent has the potential to offer T_1_ contrast due to the reduction of MnO_2_ to paramagnetic Mn^2+^ ions in the presence of GSH. T_1_-weighted MR images indicated that the contrast signal was much higher in the injected group as compared to the control group. The results show that there is a potential in the redox-activated nanoagent to enhance the efficiency of cancer detection and treatments.

The combination of photothermal therapy and redox-sensitive nanoparticle-based contrast agent was further explored by Fu and coworkers by adding manganese to copper-selenium nanoparticles. The researchers developed a novel aqueous process to coat MnO_2_ on nanomaterials to form nanomaterial-based MnO_2_ core-shell nanostructures [55]. This method can be used to combine any nanomaterial with MnO_2_, which can lead to the enhancement of the redox-responsive contrast agents by using the properties of both the materials. Cu_2−x_Se NPs were combined with MnO_2_ to form Cu_2−x_Se@MnO_2_ theranostic nanostructures, where MnO_2_ was sensitive to redox changes at tumor sites resulting in high T_1_ MR signals, and Cu_2−x_Se exhibited photothermal properties at 1064 nm to kill cancer cells. These nanostructures exhibited good blood compatibility, as only 2.2% hemolysis was seen after 4 h exposure and the molar extinction coefficient was also found to be seven times larger than that of the available Cu_9_S_5_ NPs. The redox sensitivity of the nanostructure was tested by adding dithiothreitol (DTT), which indicated a significant increase in r_1_ relaxivity from 2.26 mM^−1^.s^−1^ to 7.51 mM^−1^.s^−1^. The nanostructure was tested in vivo on nude mice with a CT26 tumor using a 1.5 T scanner, while the ratio of the pre- and post-injection signal-to-noise ratio was calculated to quantify the T_1_ signal intensity. The results indicated a 160% increase in T_1_ signal intensity at 30 min post-injection of the Cu_2−x_Se@MnO_2_ nanostructures, which remained constant for 60 min, after which a slight decrease was detected at 90 min. Despite this encouraging data, further investigation into the biocompatibility of copper-selenium should be conducted, particularly into the effect on healthy non-cancerous surrounding tissue. Extensive toxicity studies will advance this contrast agent candidate. 

Most recently, photodynamic therapy (PDT) and MR contrast were also explored. Wan and coworkers developed a GSH-unlocked Mn(III)-sealed metal-organic framework (MOF) nanosystem as a redox-activated theranostic agent for tumors [56]. The high expression of glutathione in tumor cells resists PDT and scavenges cellular ROS, limiting the clinical application of PDT. The proposed nMOF consisted of Mn(III) coordinated with a porphyrin (TCPP)-based ligand to form Mn(III)-TCPP MOFs by a one-pot method. Once internalized by the tumor cell, the Mn(III)-TCCP MOF was reduced by intracellular GSH to create glutathione disulfide, Mn(II), and free TCCP. The simultaneously activated Mn(II)-based T_1_-weighted MR imaging and TCCP-based optical imaging reduce GSH levels and controllably generate ROS. In the presence of GSH, the MOFs showed a 2.3-fold increase in T_1_ relaxation rate compared to the control, showing its potential as a GSH-activated T_1_ contrast agent. In vitro, fluorescence imaging showed that the GSH level of 4T1 cells decreased to 40% due to the reduction of the MOFs compared to groups treated with Mn(OAc)_2_ or TCPP, in which levels rose up to 90%. The MOFs were intratumorally injected into mice, and the brightness of the tumor region increased within 30 min of injection, showing a GSH-enhanced Mn-based T_1_ contrast signal. The MOFs showed no systemic toxicity in mice, even at high doses, and a hemolysis test implied biosafety and biocompatibility. It was concluded that the GSH-unlocked MOFs have the potential to increase the therapeutic effect of PDT, while avoiding negative side-effects to healthy tissue, and achieve tumor inhibition via GSH depletion. 

Despite recent advancements in photo-based therapies, researchers have also loaded redox-sensitive nanoparticle-based contrast agents with drugs that are simultaneously released with contrast agents after interactions with redox environments. Recently, Wang and coworkers presented a platelet membrane (PLTM) with biomimetic bufalin-loaded hollow MnO_2_ nanoparticles (NPs) as a tumor microenvironment (TME)-activated MR contrast agent (PLTM-HMnO_2_@BU NPs) [57]. In the tumor microenvironment, low valent ions such as Fe^2+^, Mn^2+^, and Cu^2+^ can react with H_2_O_2_ to generate hydroxyl radicals (HO^∙^) via the Fenton reaction. Drug release studies showed that tumor microenvironments such as acidic pH (5.5) and GSH (2 mM) can degrade the hollow MnO_2_ more rapidly, resulting in the highest bufalin drug release (more than 87%). For in vitro MR studies, the r_1_ value of PLTM-HMnO_2_ NPs with GSH (2 mM) was 4.525 mM^−1^s^−1^, which is significantly higher compared to the r_1_ value (0.135 mM^−1^s^−1^) of PLTM-HMnO_2_ NPs without GSH. In vivo MR studies showed that PLTM-HMnO_2_ NPs injected into the tumor area gradually increased T_1_ MR signal over time compared to nanoaprticles injected into the muscle area. This was due to the degradation of HMnO_2_ into Mn^2+^ by the overexpressed intracellular GSH at the tumor microenvironment. This study presents PLTM-HMnO_2_@BU NPs as a viable candidate for pH- and redox-sensitive dual responsive drug release MR contrast agents for tumor-specific imaging.

Preliminary GSH-sensitive nanogels containing manganese contrast agent and doxorubicin were developed by Xu and coworkers to simultaneously provide therapy and diagnostic information. The researchers formulated a redox-responsive hybrid nanosystem comprised of manganese dioxide nanoparticles and doxorubicin coated with poly(N-vinylcaprolactam) nanogels (DOX/MnO_2_@PVCL NGs) [58]. The primary role of the nanogel was to facilitate the image-guided delivery of therapeutics to the tumor microenvironment using MR and ultrasound imaging modalities. In the presence of GSH, the nanogels degraded and triggered the simultaneous release of Mn^2+^ and DOX at the tumor site for enhanced T_1_-weighted MR imaging and therapy. The efficacy of DOX/MnO_2_@PVCL NGs was investigated in neutral and acidic tumor microenvironments in the absence of GSH, resulting in 10 and 20% release of DOX over a 7-day period, respectively. When GSH was introduced, drug release improved to 89.5%. The release of Mn^2+^ was investigated using phantom T_1_-weighted MR scans. In the presence of GSH, r_1_ relaxivity was 8.33 mM^−1^s^−1^ compared to 0.04 mM^−1^s^−1^ detected in the absence of GSH. The nanogel also performed better than clinical Magnevist®, which presented an r_1_ value of 4.56 mM^−1^s^−1^ at 0.5 T. Although still in their infancy, these studies might develop into potent redox-responsive, combined diagnostic and therapeutic agents (Table 3). 

## 6. Conclusions

Contrast agents have advanced significantly in the last several years. Nanoparticle-based redox-activatable contrast agents are emerging as a subfield of interest, allowing for the possibility of visualizing dynamic processes, such as glutathione and ROS activities, in real time. This represents another take on the term “functional” MR imaging as the contrast agents allow for the imaging of a functional disease parameter or process. As the field continues to grow, researchers should consider diversifying investigations into other inflammatory diseases because most activatable agents in the pipeline are designed exclusively to detect different types of cancer, while other inflammatory diseases are less explored. The contrast agent candidates explored in this review are very exciting, but cautious optimism is required, given the small number of nanoparticle-based contrast agents that translate from bench to bedside. Researchers must endeavour to ensure that more products reach the market. Taken together, current efforts need to be sustained to ensure that redox-activatable nanoparticle-based MR contrast agents are available for relevant diseases that require soft tissue diagnostic imaging.

## Figures and Tables

**Table 1:** Glutathione-sensitive contrast agents.

MR Contrast Agent Candidate	Contrast Agent	Redox Environment	Effect Detected	Multimodal	Application
GdNPs [30]	Gadolinium	Glutathione	T_1_ Enhancement	No	In vitro detection of breast cancer cells
RANS [34]	Manganese; Iron oxide	Glutathione	T_1_ Enhancement; T_2_ Contrast	No	In vitro/in vivo imaging of gastric cancer
Fe_3_O_4_-SS-PEG Gd_2_O_3_ [33]	Gadolinium; Iron oxide	Glutathione	T_1_ Enhancement	No	In vitro detection of human kidney cancer cells
Redox-activatable fluorescence/19F-MRS/1 H-MR triple-functional probe [35]	Gadolinium	Glutathione	T_1_ Enhancement	Yes; fluorescence/^19^F-MRS/^1^H-MR	In vitro imaging of HeLa
mPEG-S-S-C16 [36]	Iron oxide	Glutathione	T_2_ Contrast	No	In vivo detection of neuroglioma in mice
MnO_2__CQDs [37]	Manganese	Glutathione; Hydrogen peroxide	T_1_ Enhancement	Yes; MR/optical probe	In vitro detection of human epithelial cells
GdNPs-Gal [38]	Gadolinium	Glutathione	T_1_ Enhancement	Yes; ^19^F-MRS/^1^H-MR	In vivo imaging of acute hepatitis in mice

**Table 2:** Reactive oxygen species (ROS)-sensitive contrast agents.

MR Contrast Agent Candidate	Contrast Agent	ROS	Effect Detected	Application
Gd-5-HT-DOTAGA [45]	Gadolinium	Hydrogen peroxide	T_1_ Enhancement	In vivo detection of inflammation in lungs of bleomycin-injured mice
Fe-PyC3A [46]	Iron	Hydrogen peroxide	T_1_ Enhancement	In vivo detection of acute and mild inflammation in mice
IPC-SPIOs [47,48]	Iron oxide	Reactive oxygen species (not specific)	T_2_ Contrast	In vitro imaging of immune cells
Fluorinated bihydrazide Gd-DOTA [49]	Gadolinium	Hypochlorous acid	T_1_ Enhancement; T_2_ Contrast	In vivo detection of murine hepatocellular carcinoma
IO-Gd NV [50]	Iron oxide; Gadolinium	Inflammatory factors	T_1_ Enhancement	In vivo detection of murine epithelial cancer cells

**Table 3:** Redox-sensitive theranostic nanoparticles.

MR Contrast Agent Candidate	Contrast Agent	Redox Environment	Effect Detected	Drug	Application
DOX@MSN-ss-GHA [53]	Gadolinium	Glutathione	T_1_ Enhancement	Doxorubicin	In vitro imaging of breast cancer cells
SiO_2_@Au@MnO_2_–DOX/Apt [54]	Manganese	Glutathione	T_1_ Enhancement	Doxorubicin; Photothermal therapy	In vivo imaging of HeLa bearing mice
Cu_2−x_Se@MnO_2_ [55]	Manganese	Tumor reducing environment	T_1_ Enhancement	Photothermal therapy	In vivo detection of murine colorectal carcinoma
Mn(III)-TCCP MOF [56]	Manganese	Glutathione	T_1_ Enhancement	Photodynamic therapy	In vitro imaging of breast cancer; In vivo imaging of cancer in BALB/c mice
PLTM-HMnO_2_ NPs [57]	Manganese	Glutathione	T_1_ Enhancement	Bufalin	In vivo detection of murine tumor
DOX/MnO_2_@PVCL NGs [58]	Manganese	Glutathione	T_1_ Enhancement	Doxorubicin	Early stage development
